# Expression of canine distemper virus receptor nectin-4 in the central nervous system of dogs

**DOI:** 10.1038/s41598-017-00375-6

**Published:** 2017-03-23

**Authors:** Watanyoo Pratakpiriya, Angeline Ping Ping Teh, Araya Radtanakatikanon, Nopadon Pirarat, Nguyen Thi Lan, Makoto Takeda, Somporn Techangamsuwan, Ryoji Yamaguchi

**Affiliations:** 1 Department of Veterinary Pathology, Faculty of Agriculture, University of Miyazaki, Miyazaki, 889-2192 Japan; 2 Department of Pathology, Faculty of Veterinary Science, Chulalongkorn University, Bangkok, 10330 Thailand; 3 STAR Diagnosis and Monitoring of Animal Pathogen, Faculty of Veterinary Science, Chulalongkorn University, Bangkok, 10330 Thailand; 4 Vietnam National University of Agriculture, Trau Quy, Gia Lam, Hanoi Vietnam; 5 Department of Virology3, National Institute of Infectious Diseases, Musashimurayama, Tokyo 208-0011 Japan

## Abstract

Canine distemper virus (CDV) exhibits lymphotropic, epitheliotropic, and neurotropic nature, and causes a severe systemic infection in susceptible animals. Initially, signaling lymphocyte activation molecule (SLAM) expressed on immune cells has been identified as a crucial cellular receptor for CDV. Currently, nectin-4 expressed in epithelia has been shown to be another receptor for CDV. Our previous study demonstrated that neurons express nectin-4 and are infected with CDV. In this study, we investigated the distribution pattern of nectin-4 in various cell types in the canine central nervous system and showed its relation to CDV infection to further clarify the pathology of disease. Histopathological, immunohistochemical and immunofluorescent analyses were done using formalin-fixed paraffin-embedded tissues of CDV-infected dogs. Dual staining of nectin-4 and CDV antigen or nectin-4 and brain cell markers was performed. Nectin-4 was detected in ependymal cells, epithelia of choroid plexus, meningeal cells, neurons, granular cells, and Purkinje’s cells. CDV antigens were detected in these nectin-4-positive cells, further suggesting contribution of nectin-4 for the CDV neurovirulence. On the other hand, astrocytes did not express nectin-4, although they were frequently infected with CDV. Since astrocytes are negative for SLAM expression, they must express an unidentified CDV receptor, which also contributes to CDV neurovirulence.

## Introduction

Canine distemper virus (CDV) is a highly virulent pathogen, which threatens various animals mainly in the order *Carnivora*. CDV belongs to the genus *Morbillivirus* of the family *Paramyxoviridae*. The virus particles possess two types of glycoprotein spikes, haemagglutinin (H) and fusion (F) proteins, on the virus envelope. The H protein binds to a cellular receptor and the F protein mediates membrane fusion. The H and F proteins play essential roles for virus entry. They are also critical for virus spread via cell-to-cell fusion. CDV transmits by inhalation of aerosol droplets or contact with respiratory tract secretions. CDV primarily replicates in immune cells, which express a specific cellular receptor, signaling lymphocyte activation molecule (SLAM, CD150)^1^. By using SLAM, CDV disseminates to lymphoid organs or tissues throughout the body, inducing lymphopenia and immunosuppression^2–7^. Recent studies demonstrated that CDV also propagates in epithelia of respiratory, urinary and gastrointestinal tracts by using nectin-4 (also known as poliovirus receptor-like protein-4 (PVRL4)) as a receptor^8^. Furthermore, CDV spreads in the central nervous system (CNS), showing neurovirulence. Our previous study demonstrated that a subset of neurons is infected with CDV and that they express nectin-4, suggesting the possible role of nectin-4 for the neurovirulence of CDV^8^. However, contribution of nectin-4 for CDV spread in the CNS still remained to be studied, because, in addition to neurons, many types of neuronal and glial cells are infected with CDV *in vivo* ^7, 9, 10^. This study aimed to reveal the distribution pattern of nectin-4 in canine tissues, especially in the CNS and to show correlation of CDV infection with the nectin-4 expression pattern of cells.

## Results

### Pathologic findings

Formalin-fixed paraffin-embedded tissues of 13 CDV-infected dogs (Nos 1–13) were used. In a previous study we have isolated Asia-1 lineage CDV strains from 6 dogs (Nos 5, 6, 9–11, 13) and Asia-4 lineage CDV strains from 3 dogs (Nos 6, 8, 12) among the 13 CDV-infected dogs (Table 1)^11^. Histological analysis by HE staining revealed CDV-associated pathological changes in various tissues, including eosinophillic intracytoplasmic or intranuclear inclusion bodies (ICIB or INIB), lymphoid depletion, and syncytial formation. Suppurative and necrotizing inflammation was observed in tissues of several dogs, suggesting secondary bacterial infections in the animals.

**Table 1:** General signalments and clinical signs of canine distemper virus (CDV) infected dogs.

Case	Breed^a^	Age	Sex^b^	Clinical signs^c^				RT-PCR^d^	CDV strains^f^
		(months)		RS	NS	GIS	SL		
No. 1	Pug	MD	M	+				N/A	N/A
No. 2	MD	3	F	+	+			N/A	N/A
No. 3	CHH	MD	M	+	+			N/A	N/A
No. 4	YT	MD	F	MD				N/A	N/A
No. 5	RT	3	M		+			F, P, H	Asia1
No. 6	MG	MD	F	MD				F, P	Asia4
No. 7	POM	2	F	+		+		F, P, H	Asia1
No. 8	MP	5 years	F	+			+	F, P	Asia4
No. 9	CHH	MD	M	MD				F, P, H	Asia1
No. 10	GR	2	F	+	+			F	Asia1
No. 11	GR	2	M	MD				F, P	Asia1
No. 12	SB	12	M	+	+	+	+	F, P, H	Asia4
No. 13	DA	17	F	+		+		H	Asia1

MD: missing data, N/A: not analyzed. ^a^Breed: CHH: Chi huahua, YT: Yorkshire terrier, RT: Rottweiler, MG: Mongrel, POM: Pomeranian, MP: Miniature pincher, GR: Golden retriever, SB: Saint Bernard, DA: Dachshund. ^b^Sex: M: male, F: female. ^c^Clinical signs: RS: respiratory disorder, NS: neurological disorder, GIS: gastrointestinal disorder, SL: skin lesion. Data for nine dogs (Nos 5–13) were reported previously (Radtanakatikanon *et al.*)^11^. ^d^Reverse transcription polymerase chain reaction (RT-PCR) showed specific bands targeting fusion (F), phosphoprotein (P) and hemagglutinin (H) genes (Radtanakatikanon *et al*.)^11^. ^f^The classified linage of nucleotide sequences of F, H, P genes were aligned with other available CDV strains in Genbank (Radtanakatikanon *et al.*)^11^.

Clinical data showed that the majority of dogs developed respiratory symptoms. Histopathological analyses revealed bronchointerstitial (Nos 2, 5–8, 11–13) and interstitial pneumonia (Nos 1, 9) with or without fibrinosuppurative inflammation. Syncytial formation was observed in bronchiolar epithelium, and both ICIB and INIB were notably seen in bronchial and bronchiolar epithelial cells. Proliferation of pulmonary alveolar macrophages was detected, and ICIB and INIB were also detected in these cells. Three out of 13 dogs (Nos 7, 12, 13) apparently developed clinical signs for gastrointestinal tract disorders (Table 1). On the other hand, the intestines in the most cases showed catarrhal enteritis and necrotic villi with infiltrative mononuclear inflammatory cells (Nos 1–2, 4–6, 8, 9, 11). ICIB was often detected in enterocytes and the lamina propria. In addition, INIB was detected in gastric glandular epithelial cells in a dog (No. 6). Lymphoid tissues or organs including spleen, lymph node, tonsil and Payer’s patch showed mild to marked lymphoid cell depletion, and INIB was detected in all lymphoid tissue samples. The urogenital lesion showed ICIB in transitional cells and epithelial cells of the renal pelvis (Nos 4, 6–8, 11). Five out of 13 dogs (Nos 2, 3, 5, 10, 12) developed clinical signs for neurological disorders (Table 1). Overtgliosis, demyelination with the presence of gitter cells, and non-suppurative polioencephalitis with mononuclear perivascular cuffing were observed in these dogs. ICIB was detected in neurons, and glia and ependymal cells.

CDV antigens were detected by IHC assay. CDV antigens were abundantly detected in various organs of all dogs (Table 2). Although analysis by HE staining showed little, if any, histopathological changes in some dogs, strong immunoreactions against CDV antigens were detected even in these dogs (Table 2). CDV positive cells were observed in both white and grey matters of each part of the brain (Table 3). CDV antigens were detected in variety of cell types in the CNS, including neurons, Purkinje’s cells, granular cells, astrocytes, ependymal cells, epithelial cells of choroid plexus, and meningeal cells (Table 3).

**Table 2:** Semi-quantitative distribution of CDV antigen in various non-central nervous system tissues by immunohistochemistry.

Case	Lymphoid organs^a^			Respiratory^b^		Stomach	Intestine^c^	Kidney^d^	UB	Others^e^
	LN	SP	TS	BE	PAM					
No. 1	NS	++	NS	+	++	NS	+	+/RP	NS	
No. 2	NS	++	NS	+	++	+++	+++	+/RP	++	+/KC
No. 3	NS	++	NS	+	++	NS	NS	++/RP	NS	
No. 4	++	++	NS	++	++	NS	+++/PP	+/RP	NS	
No. 5	+++	++	++	+	++	NS	++	++/RP	++	
No. 6	++	+++	++	+++	++	+++	+++	++/RP	NS	+/KC
No. 7	++	++	NS	++	+	NS	+/RP	+		
No. 8	NS	+++	NS	+	++	++	++	++/RP	++	+/KC
No. 9	++	+	NS	+++	++	−	++	+/RP	+	
No. 10	NS									
No. 11	NS	+++	+++	+	++	++	++	+/RP	++	
No. 12	++	+++	NS	+	+	NS	NS	+/RP	NS	++/SK
No. 13	NS	+	NS	+	++	NS				+/KC

Immunoreactivity grading: +++ marked positive, ++ moderate positive, + slight positive, −negative. NS: no sample available. ^a^Lymphoid organs: LN: Lymph node, SP: Spleen, TS: Tonsil. ^b^Respiratory: BE: Bronchial epithelium, PAM: Pulmonary alveolar macrophage. ^c^Intestine: PP: Payer’s patch. ^d^Kidney: RP: Renal pelvis epithelium. ^e^Others: KC: Kupffer cells, SK: Skin epithelium.

**Table 3:** Semi-quantitative distribution of CDV antigen in central nervous system^a^ by immunohistochemistry.

Case	Cerebrum		Cerebellum		Mid brain		Spinal cord		Meningeal cells	Ependymal cells	Choroid plexus cells
	Nu	As	Nu	As	Nu	As	Nu	As			
No. 1	−	+	−	+++	−	+	NS		−	−	−
No. 2	+	++	+	++	−	+	NS		−	−	−
No. 3	−	++	−	+++	−	++	NS		−	−	+
No. 4	+	+++	−	++	+	++	NS		−	+	−
No. 5	−	+	−	+	+	+	+	+	−	++	−
No. 6	−	++	−	+++	−	+++	−	++	+++	++	++
No. 7	+	++	−	−	−	+	−	−	−	+	−
No. 8	+	+++	+	+++	+	++	−	++	−	+	−
No. 9	−	+	−	−	−	−	−	+	−	−	−
No. 10	+++	+++	Gr	++	−	++	−	++	−	−	−
No. 11	−	+	−	−	−	+	−	+	−	++	
No. 12	−	++	−	+		+	−	−	−	−	−
No. 13	+++	++	Pu	++	++	++	NS	−	++	−	

Immunoreactivity grading: +++ marked positive, ++ moderate positive, + slight positive, − negative. NS: no sample available. ^a^Central nervous system: Nu: Neuron, As: Astrocyte, Gr: Granular cells, Pu: Purkinje’s cells.

### Nectin-4 expression in canine tissues

**Figure 1:**
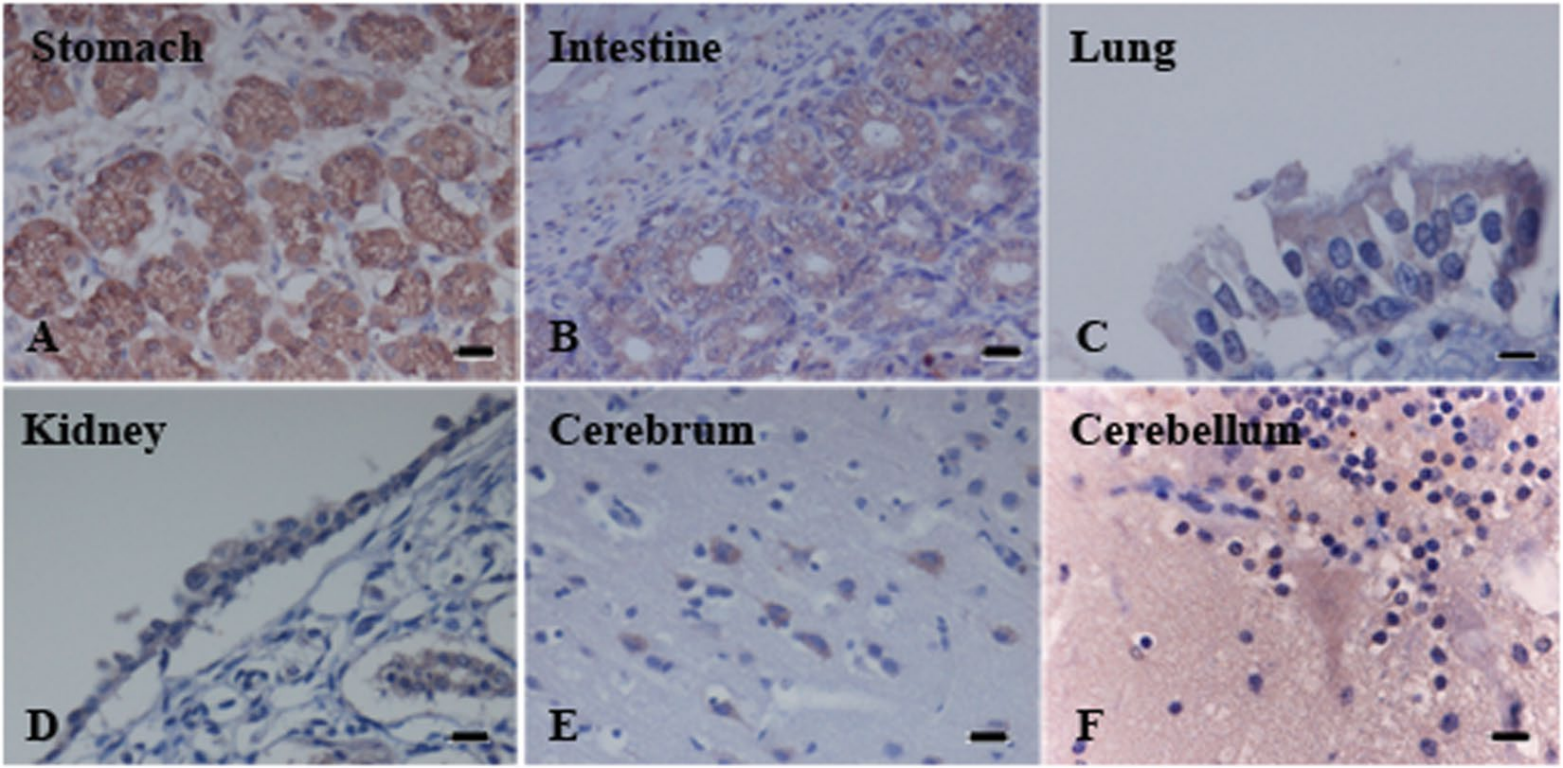
Immunohistochemistry of nectin-4 expression in normal canine tissues. Glandular epithelial cells of stomach (**A**) and intestine (**B**) showed strong immunoreactivity. Bronchial epithelial cells of lung (**C**) and pelvis epithelial cells of kidney (**D**) revealed moderate immunopositive. Neurons at cerebrum (**E**) displayed moderate to weak intensity. Purkinje’s cells at cerebellum (**F**) were positive weakly. (Bar = 20 μm).

Single staining by IHC revealed that gastric and intestinal glandular epithelial cells were strongly positive for nectin-4 (Fig. 1A,B). The epithelial cells of bronchi and bronchiole in the lung, and renal tubular and pelvis epithelial cells showed a moderate staining pattern for nectin-4 (Fig. 1C,D). Neurons at the corticomedullary junction of cerebrum were moderately or weakly positive for nectin-4 (Fig. 1E). Purkinje’s cells in cerebellum were also weakly positive for nectin-4 (Fig. 1F). Nectin-4 was undetectable in lymph nodes and spleen. In any nectin-4 positive cases, nectin-4 was distributed in the cytoplasm as well as the cell surface, but not in the nucleus (Fig. 1A–F).

**Figure 2:**
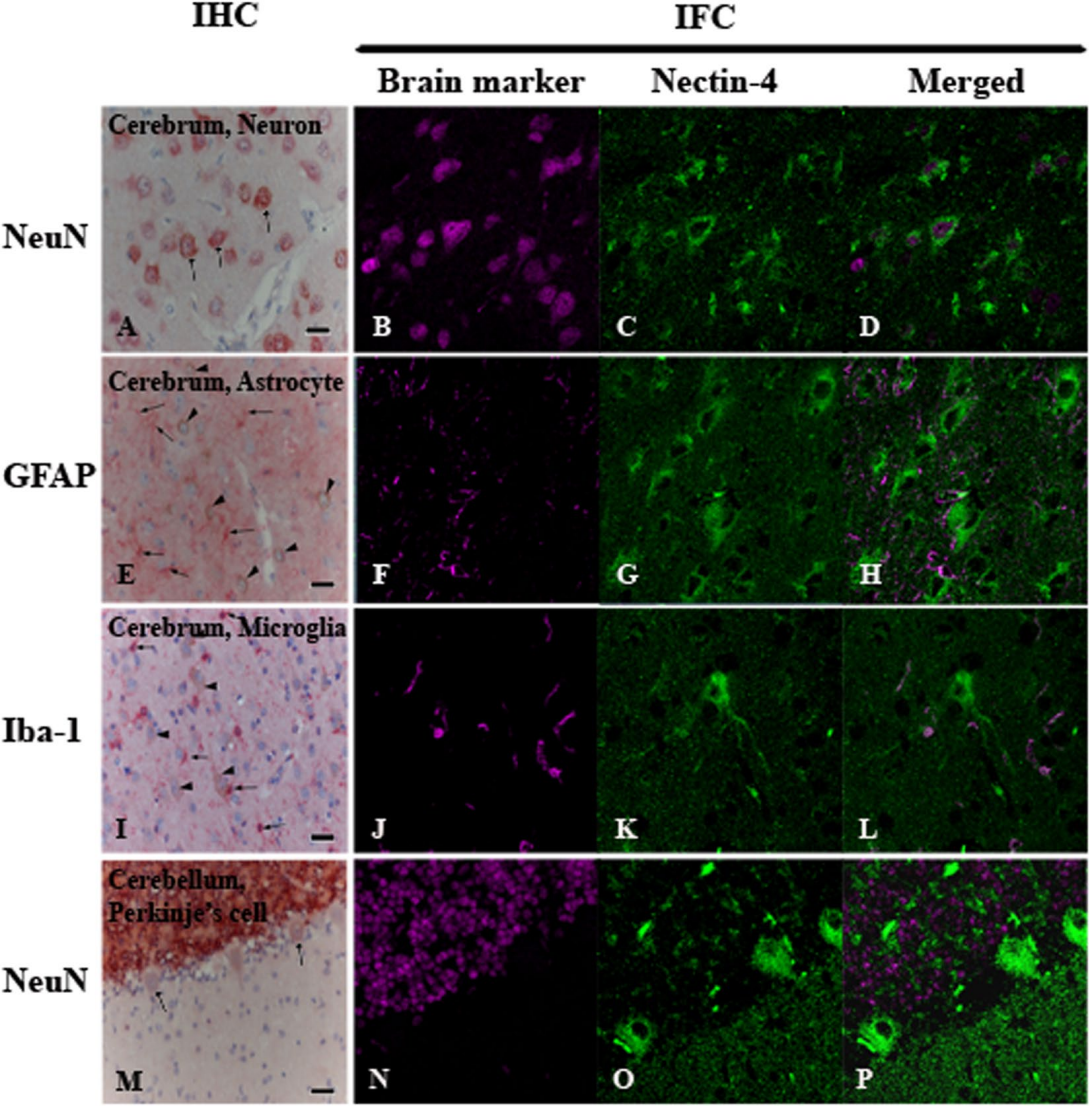
Immunohistochemistry (IHC) and immunofluorescence assay (IFA) of nectin-4 expression in normal canine brain. Nectin-4 was stained in brown (IHC) and in green (IFA) while brain markers (NeuN, GFAP, Iba-1) was labeled in red (IHC) and in magenta (IFA). Merged panel of IFA revealed the co-localization of both antigens in particular cells. Neurons at cerebrum predominately co-expressed nectin-4 and NeuN (**A**–**D**, arrows). Astrocytes at cerebrum were stained with GFAP (**E**, arrows), not nectin-4 (**E**, arrow heads indicated neurons). Microglia at cerebrum were separately immunolabeled with Iba-1 (**I**, arrows) and nectin-4 (**I**, arrow heads indicated neurons). Purkinje’s cells at cerebellum showed weakly nectin-4 positive (**M**, arrows) without NeuN staining. (IHC, Bar = 20 μm; IFA, 4,000 fold magnification).

**Figure 3:**
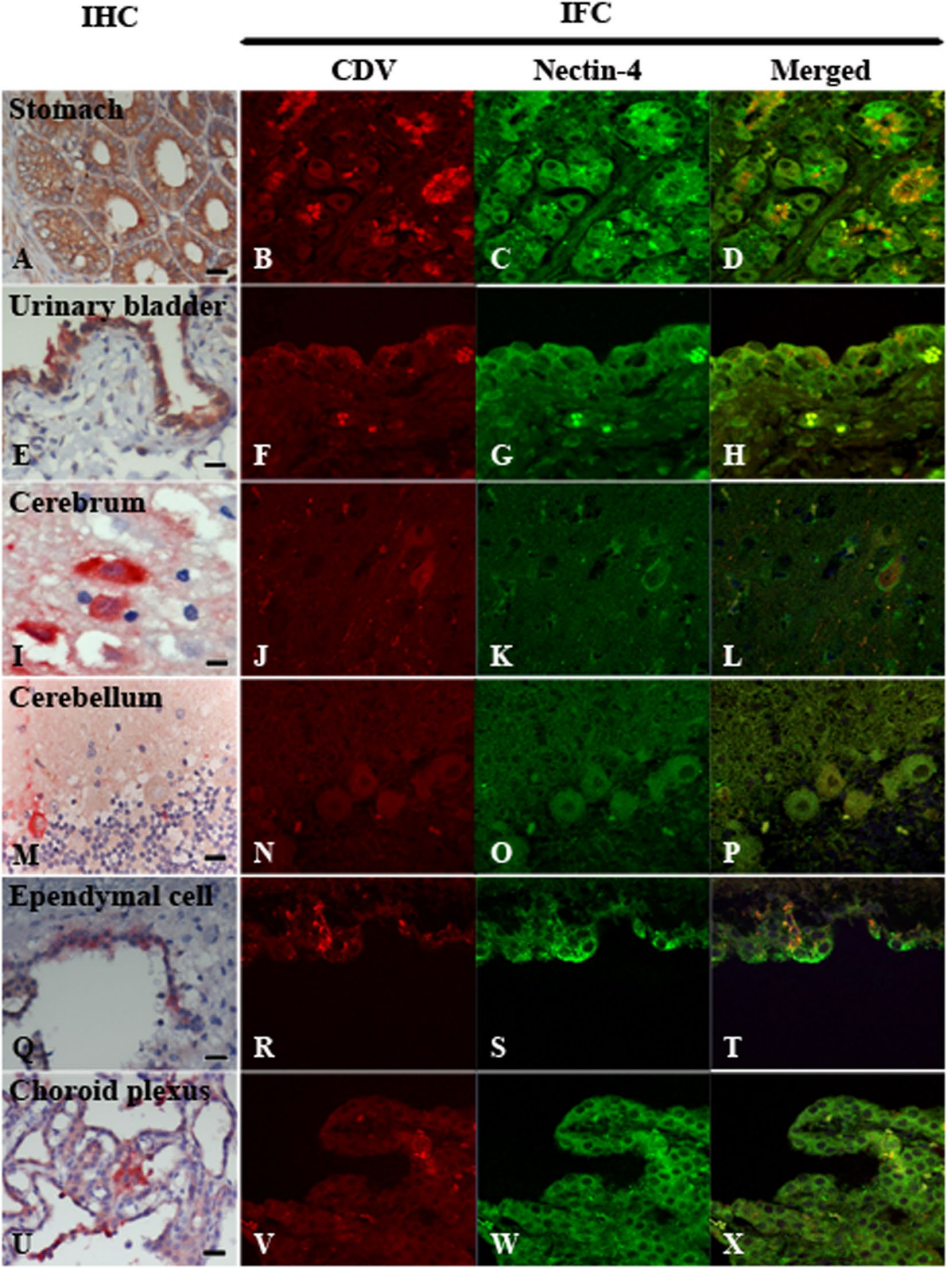
Immunohistochemistry (IHC) and immunofluorescence assay (IFA) of nectin-4 expression in canine distemper virus (CDV)-infected tissues. Nectin-4 was stained in brown (IHC) and in green (IFA) while CDV antigen was showed in red (IHC, IFA). Merged panel of IFA revealed the co-localization of both antigens in particular cells. Non-nervous tissues including gastric glandular epithelial cells of stomach (**A**; data of case No. 2) and transitional epithelial cells of urinary bladder (**E**; data of case No. 6) were strongly positive for nectin-4 and CDV antigens. The visualization of co-expression was augmented when IFA was performed (**B**–**D**,**F**–**H**). In nervous tissues, neurons in cerebrum (**I**–**L**; data of case No. 10), Purkinje’s cells in cerebellum (**M**–**P**; data of case No. 13), ependymal cells (**Q**–**T**; data of case No. 11) and epithelial cells of choroid plexus (**U**–**X**; data of case No. 6) showed the co-expression of both antigens. (IHC, Bar = 20 μm; IFA, 4,000 fold magnification).

Dual staining by IHC and IFA were performed to clarify which specific cell types in the CNS express nectin-4. In neurons NeuN was often detected together with nectin-4 (Fig. 2A–D). The nectin-4 positive rate of neurons was 58.7%. The granular layer of cerebellum was also positive for NeuN, but hardly labeled with nectin-4. Interestingly, Purkinje’s cells did not express NeuN, while they were weakly positive for nectin-4 (Fig. 2M–P). In the white matter of all sections, both NeuN and nectin-4 were only scantly visualized. Co-expression of NeuN and nectin-4 was also observed in large neurons of the mid brain and the spinal cord. By contrast, neither GFAP nor Iba-1 was co-expressed together with nectin-4 in any cell types in the CNS (Fig. 2E–H and I–L). Astrocytes were stained with GFAP but not with nectin-4 (Fig. 2E–H). The lack of nectin-4 expression in astrocytes was consistent with the previous findings by Alves *et al.* ^12^ using a primary dog brain cell culture (DBCC; mainly composed of astrocytes) and dog’s brain sections, respectively^12^. Microglias were stained with Iba-1 but not with nectin-4 (Fig. 2I–L). Ependymal cells and epithelial cells of the choroid plexus were negative for both neuronal (NeuN) and glial (GFAP and Iba-1) cell markers, while they expressed nectin-4 (Fig. 3S and W). These data clearly demonstrated that expression of nectin-4 is specific to neurons, Purkinje’s cells, ependymal cells, and epithelial cells of the choroid plexus and that astrocytes and microglias do not express nectin-4.

### Detection of CDV antigens in nectin-4 positive cells

Dual staining by IHC and IFA were performed to show correlation between nectin-4 expression and CDV infection. Nectin-4 was ubiquitously expressed in glandular epithelial cells of stomach and intestine, and CDV antigens were strongly positive in these cells (Fig. 3A–D). Also epithelial cells of the renal tubule and renal pelvis and transitional epithelial cells of urinary bladder ubiquitously expressed nectin-4, and CDV antigens were detected in these cells (Fig. 3E–H). These patterns were similar to those observed in the respiratory organs, particularly in bronchial and bronchiolar epithelia, although the nectin-4 signal intensity in the urinary and gastrointestinal tracts was greater than that in the respiratory tract. Interestingly, one sample of the tonsillar epithelial cells obtained from case No. 11 and keratinocytes of epidermis obtained from case No. 12 demonstrated co-labeling with both nectin-4 and CDV antigens (Table 2). In the CNS, dual staining pattern of both nectin-4 and CDV antigens was often seen in neurons (Fig. 3I–L). CDV-infected Purkinje’s cells (Fig. 3M–P), ependymal cells (Fig. 3Q–T), and choroid plexus cells (Fig. 3U–X) were also shown to express nectin-4. Exceptionally, astrocytes were negative for nectin-4 expression (Fig. 2G), as Alves *et al.* ^12^ demonstrated previously^12^, although they were infected with CDV (Table 3).

## Discussion

Although many aspects in the CDV pathogenicity have been obtained, molecular mechanisms of CDV spread in the CNS still remains unclear. SLAM is a principle receptor for CDV infection, and functions as a common receptor for morbilliviruses. Based on data of human and mouse tissues, the molecule is mainly expressed on dendritic cells, macrophages, immature thymocytes, and activated lymphocytes^13–16^. Interestingly, SLAM expression is up-regulated in many organs in CDV-infected dogs^17, 18^. Even in the epithelia of lung, stomach, intestine, and urinary tract, SLAM-positive cells are detected^17^, but they are likely tissue resident immune cells or infiltrated inflammatory cells. The brain is basically negative for SLAM expression, and only individual cells within blood vessel walls rarely show positive staining for SLAM^17, 19^. Despite the lack of SLAM expression, varieties of cell types, including neurons, Purkinje’s cells, granular cells, astrocytes, ependymal cells, epithelial cells of choroid plexus, and meningeal cells, are infected with CDV^20–28^. Therefore, SLAM unlikely plays a main role for the CDV spread in the CNS.

The present study confirmed our previous observations^8^ that nectin-4 is massively expressed in bronchial, bronchiolar, gastric and intestinal glandular epithelial cells, transitional epithelial cells, renal pelvis epithelia, epithelium of tonsil, and keratinocytes of epidermis. The expression pattern in epithelial tissues of dogs was similar to that in humans^12, 29^. However, our data suggested that expression pattern of nectin-4 in the CNS is greatly differ between dogs and humans. Many cell types in the CNS of dogs (neurons, granular cells, Purkinje’s cells, ependymal cells, epithelia of choroid plexus, and meningeal cells) express nectin-4, while the molecule is difficult to be detected in the human brain samples^29, 30^. In the previous study, we have detected nectin-4 in the dog brain and suggested that infection of neurons via nectin-4 contributes to neuropathogenicity of CDV^8^. The nucleotide and predicted amino acid sequences of the dog nectin-4, which has been obtained from the cerebral cortex, have been previously deposited in GenBank with the accession number AB755429^8^. Although a subset of neurons is infected with CDV, it would be evident that infection of other cell types in the CNS also contributes to the neurovirulence. Astrocytes are apparently a major target by CDV, although they do not express nectin-4^12, 19^. Demyelination, a typical pathological change due to CDV infection of the brain, coincides with CDV infection of astrocytes, while CDV antigens are poorly detected in oligodendrocytes^23–25, 27, 31^. Previous studies^21, 26^ suggested that the amounts of CDV antigens and viral RNAs in the grey matter was greater than those in the white matter. On the other hand, it was also demonstrated that CDV infection of neurons in the grey matter is not as frequent and widespread as glial infection in the white matter^20^. In any case, it is clear that CDV infection of astrocytes via an unidentified receptor also contributes to the CDV neurovirulence. Although there were variations in the distribution pattern of CDV antigens among dogs in the present study, CDV antigens were detected in the CNS of all 13 dogs. However, neurological signs were detected only in five dogs. Since many dogs have died due to secondary bacterial infections in the respiratory tract, some dogs may have died prior to neurological sign development.

Neuronal pathway of CDV infection can be explained by two mechanisms. Firstly, CDV may enter the CNS through the choroid plexus or cerebral blood vessel by using infected mononuclear immune cells. Secondly, CDV may attack via the anterograde pathway utilizing olfactory bulb as a primarily viral target organ^32^. Vandevelde *et al.* ^20^ suggested that CDV enters the CNS along the central spinal fluid (CSF) pathway, because CDV antigens are often detected in the subependymal white matter, along with the ependymal cell infection. We have also observed that ependymal cells near the choroid plexus were more frequently infected with CDV than those in other regions, suggesting that CDV spread via CSF. Nectin-4 can greatly contribute to the CDV invasion into the CNS, since meningeal cells, ependymal cells, and epithelia of choroid plexus express nectin-4. However, in this study, the expression of nectin-4 in cerebral endothelial cells was not up-regulated after CDV infection which was inconsistent with the previous study in measles virus (MV) infection^33^. Abdullah *et al.* ^33^ observed that nectin-4 was up-regulated in MV-infected human cerebral endothelial cells *in vitro*. It is suggested that MV infection of brain endothelial cells allows efficient virus production and likely allows virus invasion into the brain parenchyma in immunocompromised hosts.

Our data in the present study advanced our understanding of CDV neurovirulence. However, it is still unclear how CDV infects astrocytes. Alves *et al.* ^12^ also demonstrated that CDV infects astrocytes via SLAM and nectin-4-independent pathway. They performed the immunohistological staining and showed that nectin-4 could not be detected in the white matter of cerebellar tissues^12^, which is consistent with our findings. Besides SLAM and nectin-4, several molecules have been shown to support CDV infection. Fujita *et al.* ^34^ showed that CDV binds to a heparin sulfate molecule, and the molecule contributes to the CDV infection. CD9, a tetraspan transmembrane protein, is involved in the process of CDV induced cell-to-cell fusion, but not in virus entry^35^. Other studies also suggested that chicken embryo fibroblasts and Vero cells express unidentified CDV receptors^36, 37^. For MV, which is closely related to CDV, neurokinin-1 is shown to act as a receptor for trans-synaptic spread between neurons^38^. Identification of a CDV receptor on astrocytes is a key to further strengthen our understanding of CDV neuropathology.

In conclusion, our data in the present study further suggested that nectin-4 contributes to the CDV spread in the CNS of dogs and possibly entry into the brain via CSF. In addition, our data demonstrated that another unidentified molecule on astrocytes also plays a crucial role for the CDV neurovirulence.

## Methods

### Retrieved canine tissues

Various formalin-fixed paraffin-embedded (FFPE) tissues (brain, lung, stomach, intestine, kidney, urinary bladder, skin, lymph node) from thirteen CDV-infected dogs (Nos 1–13) were collected during submission to routine necropsy. Other two non-CDV infected dogs (Nos 14–15) were used to study the distribution of nectin-4 in a healthy condition. All dogs were retrospectively collected from Department of Veterinary Pathology, Faculty of Veterinary Science, Chulalongkorn University, Thailand during 2007–2011. The 4-μm thick sections were cut and stained with Hematoxylin and Eosin (HE). The histopathological features were determined by light microscopy. The clinical symptoms of these dogs were summarized in Table 1. All experimental protocols were approved by the Chulalongkorn University Animal Care and Use Committee (No. 11310088). All methods were performed in accordance with the relevant guidelines and regulations.

### Single staining by immunohistochemistry (IHC)

To reveal the extent of CDV infection, the FFPE sections from infected dogs were immunohistochemically processed as described previously^39^. Primary antibodies used were summarized in Supplementary Table S1. Briefly, the sections were subjected to the heat-induced antigen retrieval using autoclave in citrate buffer pH 6. Endogenous peroxidase was blocked by hydrogen peroxide (3% in methanol). Following incubation with monoclonal mouse anti-CDV antibodies (against H and F proteins; MonotopeTM, Portland, ME), the EnVision polymer (Dako, Denmark) was applied. The chromogen immunoreactivity was visualized by 3′3-diaminobenzidine (DAB; Sigma, USA) in horseradish peroxidase (HRP) system and counterstained with Meyer’s hematoxylin.

To reveal the nectin-4 positive cell types in healthy dogs, non-CDV infected FFPE canine tissues were pre-treated and visualized as mentioned above. The primary and secondary antibodies were affinity-purified polyclonal goat antibody raised against the human nectin-4 (10 μg/ml dilution, R&D system, USA) and an immune-peroxidase polymer-conjugated anti-goat antibody (Histofine® simple stain MAX–PO (G); Nichirei, Japan), respectively.

### Double staining by IHC

To elucidate the co-expression of CDV and nectin-4 in CDV-infected tissues, FFPE sections were pre-treated and incubated with the anti-CDV primary antibody as described in single staining by IHC. Subsequently, an immuno-alkaline-phosphatase polymer-conjugated anti-mouse antibody (Histofine® simple stain AP (MULTI); Nichirei, Japan) was utilized as a secondary antibody. Before visualization, sections were immersed in Tris Buffered Saline (TBS) and colorized by Fast red II in naphthol phostphate (Nichirei, Japan) using an alkaline phosphatase system. For nectin-4 staining, the same procedures as described in single staining by IHC were carried out.

To identify CDV infection of specific cell types in the CNS, samples from cerebrum, cerebellum, mid brain and spinal cord were pre-treated and incubated with a primary (rabbit polyclonal) antibody for one of the brain cell markers (NeuN, GFAP, and Iba-1) in appropriate dilutions (Supplementary Table S1). The universal immuno-alkaline-phosphatase polymer-conjugated anti-rabbit antibody (Histofine® simple stain AP(MULTI); Nichirei, Japan) and Fast red II in alkaline phosphatase system were subsequently applied. For CDV antigens staining, the procedures described in single staining by IHC were carried out.

Double staining of nectin-4 and a specific brain cell marker (NeuN, GFAP, or Iba-1) was done. The immunostaining procedures for nectin-4 and brain cell markers were described above.

### Double staining by immunofluorescence assay (IFA)

Dual staining by IFA was further performed to reveal expression patterns of nectin-4 and CDV antigens in selected FFPE tissues (brain, lung, stomach, intestine, kidney, and urinary bladder). After antigen retrieval by heat induction, the endogenous peroxidase was blocked by blocking One reagent (NacalaiTesque, Japan). Sections were first incubated with a primary antibody against CDV antigens (Supplementary Table S1) and Alexa Fluor 594-conjugated donkey anti-mouse IgG (Invitrogen, USA) as a secondary antibody. Afterwards, nectin-4 was stained with an anti-nectin-4 antibody and Alexa Fluor 488-conjugated donkey anti-goat IgG (Invitrogen, USA). The nuclei were counterstained with 4′, 6-diamidino-2-phenylindole (DAPI). The sections were covered with Vectersheild H-1500. The stained samples were observed by using the LSM 700 confocal laser scanning microscope (Carl Zeiss) and analyzed by using ZEN 2010 LSM software. The negative control was stained using healthy uninfected canine brain (Supplementary Figure S1).

To show which cell types in the CNS express nectin-4, selected CNS tissues were incubated with a primary antibody specific to one of the brain cell markers (NeuN, GFAP, or Iba-1) and Alexa Fluor 647-conjugated donkey anti-rabbit IgG as a secondary antibody (Invitrogen, USA). Double staining was performed using an anti-nectin-4 antibody and Alexa Fluor 488-conjugated donkey anti-goat IgG as mentioned above.

## Electronic supplementary material

Supplementary information
