# Supplementary material for: Expression of canine distemper virus receptor nectin-4 in the central nervous system of dogs
Source: Sci Rep. 2017 Mar 23;7:349. doi: 10.1038/s41598-017-00375-6 (PMC5428276; doi:10.1038/s41598-017-00375-6)

Expression of canine distemper virus receptor nectin-4 in the central nervous system of dogs

Watanyoo Pratakpiriya[1,2], Angeline Ping Ping Teh[1], Araya Radtanakatikanon[2], Nopadon Pirarat[2], Nguyen Thi Lan[4], Makoto Takeda[5], Somporn Techangamsuwan[2,3,*], Ryoji Yamaguchi[1,*]

[1]Department of Veterinary Pathology, Faculty of Agriculture, University of Miyazaki, Miyazaki 889-2192 Japan

[2]Department of Pathology, Faculty of Veterinary Science, Chulalongkorn University, Bangkok 10330 Thailand

[3]STAR Diagnosis and Monitoring of Animal Pathogen, Faculty of Veterinary Science, Chulalongkorn University, Bangkok 10330 Thailand

[4]National University of Agriculture, Trau Quy, Gia Lam, Hanoi, Vietnam

[5]Department of Virology3, National Institute of Infectious Diseases, Musashimurayama, Tokyo 208-0011, Japan

***Corresponding authors**

Ryoji Yamaguchi (DVM, MSc, PhD)

Department of Veterinary Pathology, Faculty of Agriculture, University of Miyazaki,Miyazaki 889-2192, Japan

Tel.: +81-985-58-7271

Fax.: +81-985-58-7272

E-Mail: a0d402u@cc.miyazaki-u.ac.jp

Somporn Techangamsuwan (DVM, MSc, PhD)

Department of Pathology, Faculty of Veterinary Science, Chulalongkorn University, Bangkok 10330 Thailand

Tel: +660-2218-9614 Fax: +660-2252-0779

E-mail: somporn62@hotmail.com

Supplementary Table S1 Summary of primary antibodies used.

Abbreviation	Antibody	Dilution	Species	Antibody	Supplier
CDV	Canine distemper virus	1:200	Mouse	Monoclonal	ViroStat®
Nectin-4	Nectin-4	1:100	Goat	Polyclonal	R&D system®
NeuN	Neuronal nuclei	1:500	Rabbit	Monoclonal	Millipore®
GFAP	Glial fibrillary acidic protein	1:2000	Rabbit	Polyclonal	Dako®
Iba-1	Ionized calcium binding adapter molecule 1	1:250	Rabbit	Polyclonal	Wako Chemicals®

Supplementary Figure S1 Immunofluorescence assay (IFA) of healthy uninfected dog's brain as negative control. Nectin-4 was stained in green while canine distemper virus (CDV) was showed in red. Merged panel of IFA could not reveal the co-localization of both antigens in particular cells (orange). Cerebrum **(A-C)**, cerebellum **(D-F)**, ependymal cells **(G-I)**, choroid plexus **(J-L)** (IFA, 4,000 fold magnification).

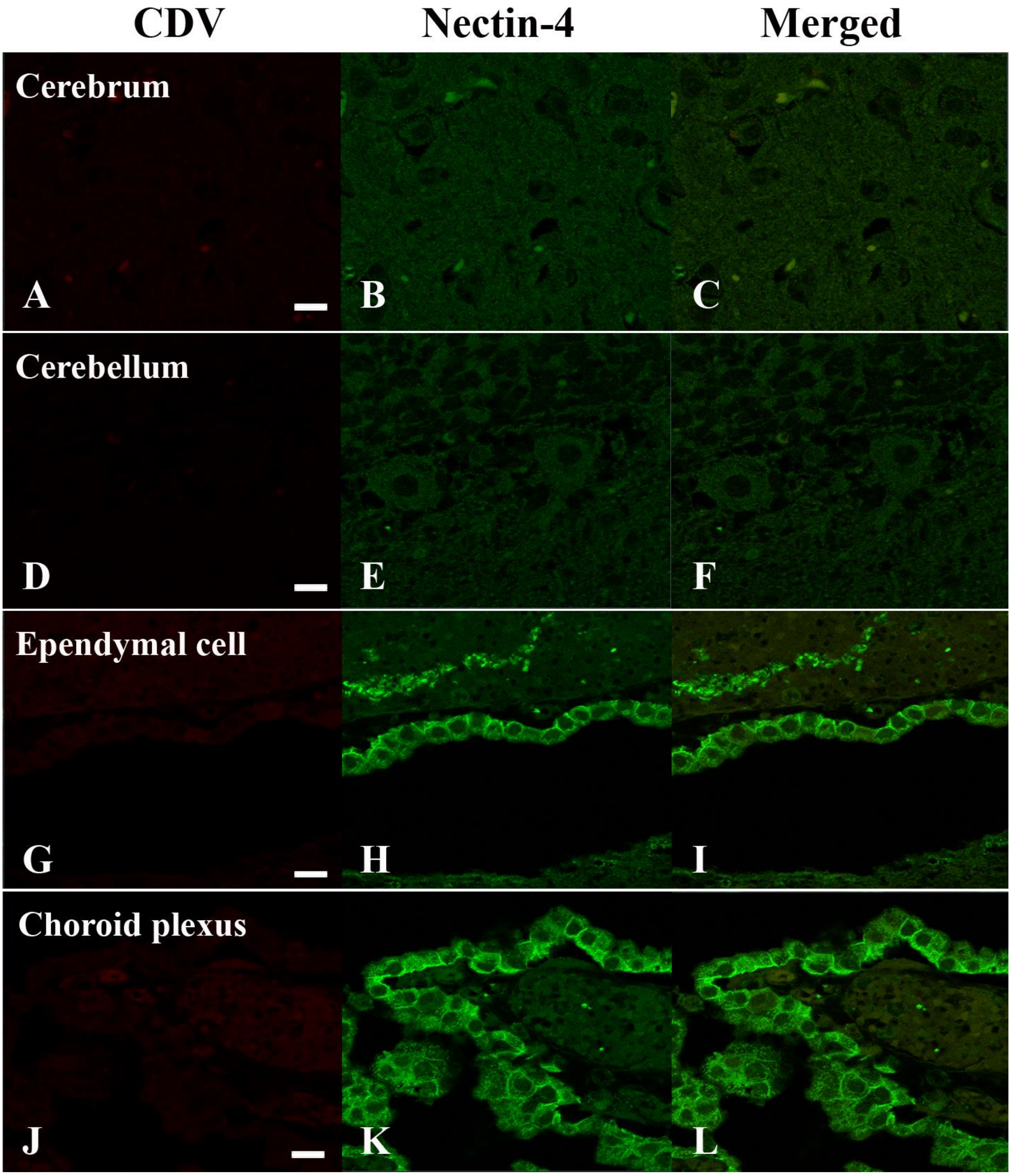

Supplement: Supplementary file 1 — Supplementary information [file 41598_2017_375_MOESM1_ESM.pdf]
